# Teaching at the Intersection of Community Engagement and Program Evaluation

**DOI:** 10.5888/pcd22.240405

**Published:** 2025-06-05

**Authors:** John M. LaVelle, Doris A. Espelien

**Affiliations:** 1 University of Minnesota Twin Cities, Minneapolis

## Abstract

The preservice education of public health professionals often includes thorough, community-engaged learning experiences. One critical element of public health work is program evaluation — an essential function for supporting evidence-based practice. However, the literature on how to prepare future public health professionals to integrate community-engaged evaluation work is lean, although lessons may be learned from the literature on service-learning. Analyzing students’ reflections in their “key learning experience” essays from an introductory program evaluation course incorporating service-learning may address this gap, helping educators identify the most effective elements of their course design and implementation. This illustrative evaluation used existing educational frameworks grounded in andragogic principles and significant learning experiences to deductively analyze 146 graduate students’ reflections on their service-learning course experience. Deductive analysis suggested that community engagement is a key element of students’ learning experience. Sixty-two (42.5%) student reflections were about community engagement, whereas 84 (57.5%) were about other topics the students found memorable. A program evaluation course that integrates service-learning may be a viable vehicle for teaching public health students about community engagement.

**Summary**

**What is already known on this topic?**

The benefits of service-learning for students, institutions, and community partners are well documented, although questions remain about what students find most effective in service-learning experiences.

**What is added by this report?**

This evaluation used established educational models and trustworthy qualitative analysis techniques to frame graduate students’ semistructured reflections about a service-learning program evaluation course.

**What are the implications for public health practice?**

Service-learning program evaluation courses may be a viable vehicle to help students engage with communities and to impart lessons about how community engagement can be done effectively across the spectrum of public health scholarship and practice.

## Introduction

Service-learning is an applied learning experience that balances the needs and interests of community partner organizations with those of the students (1,2). It is based on the premise that learning is reinforced and challenged when it is applied outside the classroom, bringing theory and practice to real-world situations. Service-learning courses in higher education are credit-bearing experiences that 1) align with specific community needs, 2) simultaneously and intentionally integrate structured reflection into the course design to discuss the course content and its professional disciplinary home, and 3) have an overarching goal of enhancing students’ sense of civic responsibility (3–5).

Program evaluation is described differently across disciplines based on the focus of the evaluation, its purpose, and the context in which it is being conducted (6). Contemporary descriptions of program evaluation position it as a process-focused discipline working with partners’ programs, policies, and interventions to help better conceptualize and describe the merit, worth, needs, and impact of their work (6,7), often with a focus on reporting, improvement, and learning for both individuals and groups. These descriptions are explicit about the central role that partner questions play in focusing inquiry processes and data collection tools, and about how evaluation is not the simplistic application of inquiry tools used without grounding in evaluation theory, ethics, and values (8,9).

## Purpose and Objectives

Several recent works address the intersection of public health and program evaluation (10–12), although the literature on how to prepare public health professionals for community-engaged evaluation work is lean. Furthermore, what elements or topics from community-engaged courses are most memorable or impactful for students is unclear from the literature, meaning that educators may not know where to invest their time and resources. The objective of this evaluation was to use established educational theories to organize and analyze students’ key learning experiences in an introductory program evaluation course that purposefully integrates service-learning into its design, implementation, and evaluation.

## Intervention Approach

Service-learning is an approved modality for applied experiences (13) in public health, and program evaluation is necessary for both supporting and refining evidence-based practice, securing funding, and engaging with communities. Both service-learning and evaluation are important for public health, although the degree to which program evaluation courses have been used to promote community engagement via service-learning is unclear. Moreover, what elements of a program evaluation course stand out most to students is unclear. Some students might find topical information about evaluation practice most meaningful, others might focus on technical information about inquiry methods, and still others might consider the service-learning elements the most memorable part of the whole-course experience. Complicating matters, each of these elements includes different combinations of factual knowledge, skills, attitudes, values, and behaviors. The lack of empirical information about what is memorable from a student perspective is problematic, as those data might be helpful as faculty engage with the course redesign process as well as demonstrating distal effects of courses and curricula.

One way to clarify these differences is to systematically collect and analyze students’ within-course reflections on their service-learning experience using previously published educational models (14). For example, principles of andragogy (15) posit that successful course design and evaluation will acknowledge and integrate elements such as students’ need to know, self-concepts, previous educational experiences, real-life orientation to learning, and an orientation toward internal motivation. Similarly, Fink’s Taxonomy of Significant Learning Experiences (16) suggests that high-impact educational experiences can be conceptually categorized across 6 dimensions: foundational knowledge (ie, learning the basic knowledge that is needed for other learning), application (ie, applying what is learned), integration (ie, connecting learning with subjects or processes), human dimensions (ie, learning about the implications of the learning for human beings), caring (ie, developing or reinforcing feelings and values), and learning how to learn. Both the andragogical and Fink frameworks have been used to analyze student reflections from program evaluation courses (17), although they have not yet been used to study what students find memorable or meaningful about community-engaged learning. Building from the assumption that structured reflections about memorable learning experiences are linked to students’ knowledge retention (18,19), the key questions are:

What proportion of students in an introductory program evaluation course described community engagement as a key takeaway from the course?
How can educational models clarify faculty learning from student reflections about their community engagement?

## Evaluation Methods

### Program context and course design

The Evaluation Studies program at University of Minnesota is housed in the College of Education and Human Development’s Department of Organizational Leadership, Policy, and Development. The program offers stand-alone master’s degrees, doctoral degrees, and certificates of advanced study while also serving as a minor area of study for students across the institution. Both the stand-alone and the minor degrees launch from the semester-long 3-credit Introduction to Program Evaluation course (OLPD 5501), which was designed to align with the American Evaluation Association’s *Competencies* framework (20). This course offers introductory evaluation concepts alongside practical evaluation experience (21) through community engagement, interwoven with course content and educational activities.

The educational activities in the course include a reflective final examination and the development of an evaluation plan for a community partner of each student’s choosing. Topics required in the evaluation plan are aligned with the major course topics, such as information about the program context; the evaluation rationale; a list and description of interested partners and constituents; a visual and narrative logic model; key evaluation questions; evaluation design information (eg, study design, sampling, inquiry tools, and analysis plans); and a management plan, budget, and list of constraints. Examples of project foci include disability services, supports for transition-age youth with disabilities (young people transitioning from adolescence to adulthood), immersive nature-based educational experiences, health disparities–reduction initiatives, K-12 student nutrition and health programs, and mental health services. Students are expected to share their developing work with their community partners in at least 4 synchronous meetings throughout the semester and to present their final proposal at the end of the semester. As part of their involvement in the course, community partners are asked to share background information about their program design and implementation and to be available to give feedback on students’ logic models, activity descriptions, outcome definitions, and proposed data collection strategies.

### Participants

Participants were 146 graduate students in the first author’s Introduction to Program Evaluation course at University of Minnesota since fall 2017. Course sizes ranged from 6 (pandemic year) to 34 students, and courses were taught in-person using a synchronous format with required engagement with community partners; the number of community partner projects per course session ranged from 2 to 9 and varied as a function of course enrollment. Students were working toward their graduate degrees in fields including public health, medicine, pharmacy, public policy, education, and environmental sciences.

### Assessments and measures

Service-learning and active reflection were built into each course meeting session and culminated in a reflective final examination that included an anticipatory reflection prompt (22): “In one page, describe your key learning experience or your ‘a-ha!’ moment from this course, why it was meaningful, and why you think it will be memorable.”

### Data analysis

We first inductively organized the data based on whether the reflections discussed communities and community engagement, then deductively analyzed the data at the sentence-level of analysis (23,24) based on andragogical principles (15) and Fink’s Taxonomy of Significant Learning (16). To establish the trustworthiness of our analysis and interpretation, we read the reflections independently to see if they should be included in the analysis; we then analyzed the included data independently and followed this with discussion to reconcile each other’s interpretation and application of the frameworks. Through these discussions, we triangulated the data with a focus on trustworthiness, replicability, and data saturation before selecting illustrative quotes. Attributions for individual quotes were scrambled for anonymity. Data were not disaggregated by respondent demographics or disciplinary home. This evaluation was declared exempt by University of Minnesota’s institutional review board.

## Results

The results of this illustrative evaluation show ways in which community engagement sparked students’ key learning experiences. Of the 146 students who provided reflections, 84 (57.5%) wrote about noncommunity aspects of the course, including 3 students who wrote about other topics but did not elaborate in ways that allowed for categorization. The other 62 (42.5%) wrote about topics related to community engagement and how the course content helped them better work with their community partners. The analysis showed that student reflections could be categorized and analyzed using both andragogical principles and Fink’s taxonomy to determine what was memorable for the students (Table), that the educational frameworks are helpful for organizing students’ reflections, and that each component of each framework is reflected in their discussions at least one time.

**Table:** Illustrations of Students’ Reflective Data, Organized by Educational Taxonomies

Construct	Definition^a^	Illustrative quotes
**Andragogical principles**		

Need to know	“Adults need to know why they need to learn something before undertaking to learn it” (p. 64)	Honestly, I did not understand the purpose of doing the key definition exercise until after our group completed the exercise and later talked with our client. (X.B.)

Self-concept	“Adults have a self-concept of being responsible for their own decisions” (p. 64)	While I knew that I was entering into this semester with an “informed novice’s” understanding of how evaluations take place, I also knew that my attention to detail is not as good as it should be and that I would need support. . . . Through my teammates [for the community project], I witnessed another demonstration that myriad skills are needed to conduct an evaluation successfully. (H.Q.) I am a very hands-on learner, so having the ability to work with a client and create a plan was helpful to apply the concepts that we discussed in class. (E.O.)

Previous experience	“Adults come into an educational experience with both a greater volume and different quality of experience from that of youths” (p. 65)	[Reflecting upon the ethical responsibility to remember that each data point is a human being] I feel like this really strikes a chord with me because I have been in positions in which my job requires me to gather data, but this data has never been used. It is nice to feel validated in the sense of unfairness I have felt with this. It reinforces my belief that without being intentional with evaluations, and how organizations go about gathering data, the more likely that the outcome will be more harmful than helpful. (A.G.) This past semester has definitely been a learning curve for me since I had never encountered any material in regards to knowing the methods and principles behind evaluation. . . . I believed that evaluation was only data-driven, but now I realize that it is more of telling a story. (E.O.) I had always worked under the assumption that while evaluators have experiences and bias that impact how they work, the process of evaluation would be the same for all evaluators. I truly had thought that there was a guiding process (step 1, step 2, etc) that all experienced evaluators would be sure to follow. (Y.P.)

Readiness to learn	“Adults become ready . . . in order to cope effectively with their real-life situations” (p. 67); “adults are motivated to learn [if] . . . they perceive the learning will help them perform tasks or deal with problems they confront in their life situations” (p. 67)	The whole experience of working with a client throughout the class does add to my confidence of working on evaluation assignments. (K.Z.) Overall, I think these communication skills will be greatly helpful for future evaluation work, and any other work, truly. [Through the community project] I was able to practice finding the “gaps in logic,” identifying assumptions, and managing how to directly and kindly seek clarification as needed. (O.G.) Our greatest disagreements, sometimes very heated, may come from fundamental misunderstandings of how concepts are defined rather than disagreements about the ideas that are derived from them. I noticed this phenomenon in my personal life. (D.Q.)

Task-centered or problem-centered	In contrast to children’s and youths’ subject-centered orientation to learning, adults are life-centered (or task-centered or problem-centered) (p. 67)	[During the service-learning project, the stakeholder] kept on adding more dimensions, which practically may not be in sync with [program]. He was seriously contemplating a larger vision and was seeking our help to make it part of our plan. When we started putting everything into the logic model, we were all over the place not able to find a “logic” of flow. I was in a dilemma of whether to share my impression with [program client]. One group member had a strong opinion that we should be doing what our client expects us to do. . . . During presentations and interactions with [instructor name], I asked this dilemma in different ways, and thankfully I was able to come to terms with how to approach this issue. (Q.E.)

Motivation to learn	“Adults are responsive to external motivators . . . but the most potent motivators are internal pressures” (p. 68).	I have been working to document and process the work we do at [organization name] and think about the ways each activity does or does not support our overall goal. . . . I now feel better equipped to work alongside young people to both create a logic model and develop a program evaluation from that logic model. (Z.I.)

**Significant learning experiences**		

Foundational knowledge	Refers to the students’ ability to understand and remember specific information and ideas.	Before the course, I had an impression that the process of evaluation starts once a program or a project comes to an end. However, it was interesting to learn that evaluation is a continuous process, and we can get started with the process of evaluation right at the start of the program. I realized how one need not wait [until] the end of the program to have a logic model prepared or list down the key definitions that the evaluation would revolve around. (K.Z.)

Application	Application learning allows other kinds of learning to become useful.	Being nimble and having flexibility as a team (and also asking the right questions, getting clarification, and listening to hear) allowed us to create a proposal that was more meaningful and useful to the client. (L.I.) When we began the [practical application] exercise, I saw the company information as Swiss cheese, and we use questions to fill in the holes of our knowledge of the company’s programming. . . . After listening to the questions of the other groups and listening to [instructor] give his responses, I learned that what I first believed to be Swiss cheese was in fact a sponge. Instead of a few simple questions there was a plethora of unknown variables and aspects to be considered. . . . I learned to look for the strings, the hidden connections. (G.F.) [The service-learning project] reiterated how much goes into a plan; it seems like the actual evaluation is the easiest part. (E.O.) One of the key learning insights I’m taking with me is that quality evaluations executed correctly are expensive! [Despite the evaluation being straightforward, the questions uncomplicated, and a substantial portion of our time being pro bono] the total cost came to $25,000. . . . [Program] has no money to pay for the evaluation, and further, they want us to look for money to pay for it. (W.L.)

Integration	The act of making connections gives learners a new form of power, especially intellectual power.	[My project team] was very different in its disciplinary composition. It consisted of folks from social work, development practice, public health, and evaluation, which showed me that evaluation is not only nimble enough to be applied in different sectors but can be implemented successfully when the team comes together toward a common goal that they personally may not know much about, in this case, [name of program]. (X.K.) In taking a course on ecology, we discussed evaluation of ecosystem services and had to define what was an ecosystem service and what made it a service. I knew from taking this course in evaluation that it wasn’t going to be as easy as it looked on the surface, and the definitions we came up with through the class were all slightly or drastically different. It really showed the importance of definitions and how that can completely drive an evaluation. (E.E.)

Human dimension	This kind of learning informs students about the human significance of what they are learning.	When one of our colleagues asked our group if we had discussed the budget with our client, my reaction was to laugh. This was an extremely teachable moment as the budget sets a parameter and constraints on the evaluation design. If we had discussed the budget with our primary stakeholder while we were in the process of designing the data collection and analysis methods we could have put together a design that was more appropriate for the needs of our primary stakeholders. (D.Q.) We had to think about whether or not stakeholders at [organization name] would be open to considering more practical goals. (S.I.Z.) What [person’s name] showed me was that if the stage is properly set for the evaluation and people do not feel threatened, the insights from program staff can be what makes the difference between an evaluation that will land in a file drawer and an evaluation that can fundamentally reshape a program for the better. (E.G.)

Caring	When students care about something, they then have the energy they need to learn more about it and to make it a part of their lives.	My main takeaway from the course is that evaluation is a slow process that cannot be rushed. At the beginning of the course, I wanted to breeze through the evaluation process to find tangible solutions for the client. After the initial client visit, I thought I knew the exact changes that were necessary to make this employee career center effective. However, I didn’t realize the biases I was bringing into the process. I failed to truly listen to the client because I based their needs on my own assumptions. (W.I.) Our group project was designed with a client who was not terribly invested in our evaluation. We were able to design the evaluation around the questions that we — as students and [city] residents, and thus as stakeholders — felt were relevant. But we were able to ask the relevant questions and determine that relevancy with accuracy largely because of our role as stakeholders. (T.Z.)

Learning how to learn	This kind of learning enables students to continue learning in the future and to do so with greater effectiveness.	My biggest takeaway from this course has been realizing how big of a role an evaluator’s subjectivity can play in any given evaluation. . . . [I learned] that even coding systems can be subjective, and I realized the importance of working in evaluation teams and preserving audit trails. (L.X.) [The definitions exercise was] memorable because I found it to be useful in other areas of my studies, as well as in our own evaluation. (E.E.) I really enjoyed the service-learning component of this class. At first I found the idea of approaching organizations to be intimidating, but I’ve been learning . . . that if you tell someone you are a graduate student and you wish to learn from them, they usually help you out. I’m sure the approach of asking to learn from someone can also be used in the future, even after grad school! (F.P.)

a Definitions from Knowles et al (15) and Fink (16).

## Implications for Public Health

In conjunction with other scholarship (25,26), this evaluation showed ways in which evaluation courses that incorporate a service-learning framework might be helpful vehicles for promoting students’ engagement with communities. The proportion of students (42.5%) who described community engagement in their reflective essay was noteworthy. The community engagement aspect of the course was memorable for many, suggesting that students wanted to engage with program evaluation and their primary discipline simultaneously in ways that are relevant and applicable to them. We imagine that replication studies would find similar results (27) and encourage educators to find creative ways of engaging with communities and applying their disciplinary expertise while being mindful of the risks (eg, power imbalances between the institution and partners, accidentally reinforcing student or faculty stereotypes of service providers or their constituents) (28). Analyzing student reflection data with an emphasis on understanding meaningful learning experiences about community engagement allowed us to explore the many ways in which students make sense of their own learning; using the existing frameworks to organize the students’ reflections made those reflections manageable and interpretable.

One surprising element of the analysis was how students’ reflections lent themselves much more to the Significant Learning framework (16) than to the andragogy framework (15), although this may be an artifact of how the reflective prompt was framed. Reflections consistently incorporated elements from both models, and in some cases, the frameworks seemed to work in conjunction with each other. That is, in reflections where elements from both models were coded, it was common to see elements of the andragogy framework described first, followed by an elaboration drawn from the significant learning experience elements (such as the reflection from X.K.) (Table). We interpreted this to mean that the students described their key learning experience (ie, processes) by first reflecting on where they were before they began to engage with the material (ie, inputs) and then discussing the implications (ie, anticipated outcomes) for the inputs plus the processes.

The process and results of this evaluation suggest the utility of Fink’s taxonomy as an evaluative tool for course improvement, which challenges another case study on applying theory in practice (29) wherein the andragogical framework is more prevalent. This may be a result of the different course foci (ie, evaluation theory vs evaluation practice), although it might also reflect the idea that andragogical frameworks (15) place a greater emphasis on course design and implementation (ie, inputs), and Significant Learning (16) places greater illustrative power on course effects (ie, outcomes). We interpreted this to mean that both frameworks have value for organizing and interpreting qualitative information about student experiences.

As educators, we appreciated these students’ reflective data because they are unvarnished reactions to things students felt were important and gave us insights that we can intentionally integrate into future course iterations. Examples of what future students might find helpful could include being more explicit about the rationale that goes into each element of the course design and deliverables, doing more coaching about processes and techniques for reaching out to potential community partners, and perhaps more systematically inviting community partners to join the final course meeting for the students’ final presentations.

### Limitations and next steps

This evaluation has several limitations. First, the student reflections were drawn exclusively from courses taught by the first author, potentially reflecting the instructor’s emphasis on community engagement (30,31). Reflections from students in courses taught by other faculty may highlight different learning experiences. Second, as an illustrative evaluation, this evaluation did not seek to measure the relative prevalence of theoretical components or test the data’s alignment with existing theories. The evaluation reflected both andragogical principles and Fink’s Taxonomy of Significant Learning, but the data analysis processes were not used to clarify the interplay between them. Future research should address this gap and examine how these constricts influence long-term knowledge retention and application, particularly for public health students (18,27). Third, the evaluation did not disaggregate the data based on students’ academic discipline, and exploring whether systematic differences exist in student reflections based on their disciplinary focus will be important future work. Lastly, the evaluation did not address how the community partners benefited from the collaboration. Anecdotal feedback was positive, but systematic assessment and evaluation is necessary to assess these outcomes.

### Implications and conclusion

This evaluation demonstrated that integrating service-learning into program evaluation courses can effectively prepare community-engaged public health professionals. This approach strengthens students’ practical evaluation skills and deepens their conceptualization of community engagement — a core element of public health practice. Future research should investigate the long-term effects on students’ professional development and the tangible benefits for community partners. Furthermore, evidence is needed to support the claim that exploring what students find memorable about community engagement leads to meaningful learning outcomes. Providing this evidence will strengthen the rationale for using reflective analysis in public health education. Expanding service-learning opportunities in public health education can better equip students to apply evidence-based evaluation methods in real-world settings, advancing more effective and equitable public health initiatives.
